# Human Papillomavirus Infection Correlates with Inflammatory Stat3 Signaling Activity and IL-17 Level in Patients with Colorectal Cancer

**DOI:** 10.1371/journal.pone.0118391

**Published:** 2015-02-23

**Authors:** Yi Xin Li, Lei Zhang, Dilixia Simayi, Nan Zhang, Lin Tao, Lan Yang, Jin Zhao, Yun Zhao Chen, Feng Li, Wen Jie Zhang

**Affiliations:** 1 Department of Pathology/the Key Laboratories for Xinjiang Endemic and Ethnic Diseases, Shihezi University School of Medicine, Shihezi, Xinjiang, China; 2 Department of Pathology, the First Affiliated Hospital, Shihezi University School of Medicine, Shihezi, Xinjiang, China; 3 Changzhou Children’s Hospital, Changzhou, Jiangsu, China; 4 Key Laboratory of Chinese Ministry of Education, Xinjiang Medical University, Urumqi, Xinjiang, China; National Institute of Health - National Cancer Institute, UNITED STATES

## Abstract

**Background:**

Colorectal cancer (CRC) is a major burden of public health and healthcare worldwide. Microbiota has been suggested in promoting chronic inflammation in the intestine which, in turn, promotes tumor development. This study focuses on possible correlations of human papillomavirus (HPV) infection with proinflammatory Stat3 signaling activities and the resulting levels of its downstream proinflammatory cytokine IL-17 in CRC patients.

**Methods:**

HPV was examined using HPV Genotyping Chip technology and constitutively active Stat3 (p-Stat3) and IL-17 levels were tested using immunohistochemistry (IHC) in paraffin-embedded cancerous and adjacent normal tissues (ANT) from a cohort of 95 CRC patients. Correlation analyses were performed between HPV infection and clinicopathological characteristics, Stat3 activities and IL-17 levels among these CRC patients.

**Results:**

Three major findings were observed: (1) HPV infection existed in a high rate of CRC cases (48.4%, 46/95), of which 45 cases (45/46, 97.8%) were high-risk HPV16-positive and only one case was HPV53-positive. (2) HPV infection correlated with poorer clinical stages (III+IV) of CRC. (3) HPV infection strongly correlated with both constitutively higher Stat3 activities (P<0.01) and higher IL-17 levels (P<0.01) only in CRC tissues but not in ANT tissues.

**Conclusions:**

HPV infection is common in CRC patients suggesting potentially preventive effectiveness of HPV vaccination among high-risk young individuals. We have for the first time revealed a tri-lateral relationship among HPV infection, constitutive Stat3 activity and IL-17 level, whose collaborative act may orchestrate a proinflammatory microenvironment in the colorectum that, in turn, may promote carcinogenesis and possibly facilitate progression of CRC.

## Introduction

Colorectal cancer (CRC) is the third most common cancer globally following lung cancer and breast cancer, with incidence, mortality and 5-year prevalence accounting for 9.7%, 8.5% and 10.9%, respectively, among all cancers according to GLOBOCAN 2012 [1]. The CRC death rate has declined worldwide in the past few years thanks to screening programs established in many countries and demonstrable impact of risk-reductions and improved treatments [2,3].

The current paradigm is that most solid tumors including CRC are linked to chronic inflammation, directly or indirectly [4]. Among many microorganisms studied, viral infections are suggested in cancer development especially that caused by human papillomavirus (HPV). Viral infections by high-risk HPV subtypes, such as HPV16 and HPV18, are causal to the development of cervical, anal and genital cancers. Recently, HPV16/18 infections have been reported to be associated with oropharyngeal cancer [5,6]. In the last few years, HPV infection has been shown to be associated with colorectal cancer [7,8].

Due to various inflammatory cells and cytokines present in tumor microenvironment, tumors have been referred to as “wounds that do not heal” [9]. Signal transducer and activator of transcription 3 (Stat3) is a critical signaling pathway that is involved in the formation of tumor microenvironment through regulating downstream proinflammatory cytokines and factors promoting tumor growth and progression [10]. Phosphorylated Stat3 (p-Stat3) is the active form of Stat3 that can be detected in various cancers including colorectal cancer [11]. Among the downstream molecules induced by Stat3 activation, IL-17 is an essential proinflammatory cytokine secreted by T-helper IL-17-producing (Th17) cells which are produced by CD4^+^ T cells under the induction of TGF-β. Recent studies have suggested that IL-17 plays a dual role in serving either as a promoter or antitumor factor depending upon cancer models [12]. In CRC, a large body of studies demonstrates that IL-17 acts as a promoter rather than an antitumor factor in tumor initiation and progression [4,13,14]. Constitutively active p-Stat3 can regulate the differentiation and maturation of Th cells to secret IL-17, which, in turn, positively feeds back to Stat3 signaling, inducing more IL-17 to be expressed. Stat3 signaling plays a role in promoting tumor via inducing the formation of tumor blood vessels, facilitating the accumulation of neutrophils, enhancing tumor cells’ ability to resist apoptosis, and constantly expanding the local inflammatory response [4]. On the other hand, IL-17 promotes the carcinogenesis primarily by arousing the activation of Stat3 signaling pathway to induce resistance to apoptosis and promote angiogenesis by which facilitate tumor growth and progression [15].

Bacterial infections have been shown to collaborate with Stat3 signaling pathway to induce CRC [4]. It is not known, however, whether viral infections may also be able to act in the same fashion as bacteria. This study focuses on possible correlations of HPV infection with proinflammatory Stat3 signaling activity and its downstream IL-17 cytokine in CRC. We have examined the hypothesis that, similar to bacterial infections, chronic HPV infection may also be able to collaborate with Stat3 signaling, triggering an inflammatory response that facilitates the formation of a microenvironment favorable for carcinogenesis and/or cancer progression of CRC. By examining a cohort of 95 CRC patients’ tissues and their adjacent normal tissues (ANT), we have obtained three major findings: (1) HPV infection exists in almost a half of CRC cases, of which 97.8% are infected with high-risk HPV16 type; (2) HPV infection correlates with poorer clinical stages of CRC; and (3) HPV infection strongly correlates with constitutive activities of Stat3 and its downstream IL-17 levels in CRC tissues. These observations have a potential implication underlying an HPV-Stat3 mechanism in the pathogenesis of CRC and, a possible preventive application of HPV vaccination among high-risk young individuals at least in regions with prevalent HPV-associated CRC.

## Methods

### Ethics Statement

This study was approved by the Institutional Ethics Review Board (IERB) at our First Affiliated Hospital of Shihezi University School of Medicine (IERB No. SHZ2010LL02). The IERB waived the need of patients’ consent due to anonymous analyses of the data and standard university hospital guidelines in accordance with the Declaration of Helsinki including confidentiality and anonymity were followed in the handling and publication of patients’ tissues.

### Patients

A total of 235 surgically resected and paraffin-embedded archival human tissues was obtained, including 95 CRC tissues and 90 paired ANT tissues, and 50 colorectal adenoma (CRA) tissues from the Department of Pathology, the First Affiliated Hospital of Shihezi University School of Medicine dated from 2005 to 2007. All selected tissues were based on histopathological diagnoses and reconfirmed for diagnoses before experimentation. These patients were originally selected because of both their available clinical information and sufficient tissue size for experimental analyses (including repeats). CRC patients were aged from 33 to 85 years (with a median age of 66 years) including 43 cases of colon cancer (with 41 paired ANTs available) and 52 cases of rectal cancer (with 49 paired ANTs available). The 5 CRC patients without paired ANTs remained in the study in order to gain statistical power in clinical analyses with several sub-groupings. These CRC patients did not receive preoperative chemotherapy or chemo-radiotherapy and their clinicopathological characteristics were summarized.

Due to consumption of tissues in prior studies and required large size of tissues for DNA extraction and subsequent HPV testing, only 50 paired ANT tissues were available that satisfied the selection criterion of 5 cm away from the edge of cancer. For DNA extraction and HPV testing, therefore, we were only able to test these available 50 paired ANT tissues which were randomly distributed among paired CRC tissues. In addition, we also tested 50 unrelated CRA tissues, a common non-cancerous disease of the digestive system, for HPV infection to investigate whether there was any difference between CRC and CRA, of which CRA tissues served as a different control group in terms of HPV infection.

### Detection of HPV Infection Using Gene Chip Technology

Genomic DNA was extracted from tissues using a DNA extraction kit following manufacturer’s protocols (QIAGEN, Germany). DNA specimens were then coded without identification of cancer or non-cancerous diagnoses and sent to a specialty DNA genotyping laboratory (Yaneng Bioscience Co., Ltd, Shenzhen, China, http://www.yanengbio.com/en/home2.asp) for HPV testing. The quality and integrity of extracted DNA was tested by PCR using β-actin gene as an internal control (forward primer: 5’-CTTAGTTGCGTTACACCCTT-3’ and reverse primer: 5’- TGTCACCTTCACCGTTCC-3’). A 155 bp PCR product of β-actin gene was detected by electrophoresis on 2% agarose gel.

At the request of Yaneng Bioscience Co., the following precautions were taken to avoid possible cross-contamination during the process: (1) Single-use disposables were employed in DNA extraction and subsequent HPV testing; (2) From the first batch of extracted DNA and HPV test results, we selected one negative HPV control (an ANT sample with large size of tissue) and one positive HPV control (a CRC sample with large size of tissue) to be included in every batch of DNA extraction (20 samples per batch); (3) In each batch of DNA extraction, equal numbers of CRC tissues (10 per batch) and ANT or CRA tissues (10 per batch) were included for DNA extraction; and (4) These control DNAs were tested for HPV together with the same batch of DNA extracts. Throughout the entire DNA extraction and HPV testing, we noticed two batches of DNA extracts among which the positive control did not show positivity for HPV and these two batches of 40 tissues were then re-extracted for DNA and re-tested for HPV until the positive HPV control was positive for HPV test. However, we did not observe the negative HPV control turned to be positive through DNA extraction and HPV testing.

HPV genotyping was performed using HPV Genotyping Kit, a clinical diagnostic product of Yaneng Bioscience Co. (Chinese State FDA approval no. 2008-340099) in the Specialty HPV Genotyping Laboratory within Yaneng Bioscience Co. which had been chosen by the International Agency for Research on Cancer (IARC, World Health Organization) to perform the Prevalence Surveys of HPV Infection and Cervical Neoplasia in China. PCR reaction mixture (25 μl) contained 5 μl of genomic DNA, 20 μl of PCR reaction buffer. Cycling conditions were as follows: Initial denaturation at 95°C for 5 min, then 40 cycles at 94°C for 30 s, 42°C for 90 s, 72°C for 30 s, and a final extension at 72°C for 5 min. A positive HPV control DNA was placed on each hybridization blot (S1 Fig.). The reverse line blot method (RLB) was used on all specimens with amplifiable DNA and the hybridization conditions were described previously [16,17]. PCR products were manually loaded onto the chip blots and hybridization procedure was performed by automation. The blots were initially read automatically and checked manually before reporting (S1 Fig.). The HPV Genotyping Kit was able to detect 23 HPV subtypes including 18 high-risk HPV (HR-HPV) types (HPV16, 18, 31, 33, 35, 39, 45, 51, 52, 53, 56, 58, 59, 66, 68, 73, 83, and MM4) and 5 low-risk HPV (LR-HPV) types (HPV 6, 11, 42, 43, and 44) (S1 Fig.). Each DNA specimen was triplicated in 3 gradient concentrations on genotyping chip blots and the positive coincidence rate was 100%. This HPV genotyping test has a sensitivity of detecting 103 copies of HPV DNA/ml and a specificity of 99% based on the company’s specifications. HPV test results were returned to us and decoded to match patients’ identifications before analysis.

### Detection of Active p-Stat3 and Expressed IL-17 Using Immunohistochemistry (IHC) Assay

Paraffin-embedded tissues were sectioned in 4-μm slices and IHC analyses were carried out using streptavidin peroxidase method (SP kit, Zhongshan Golden Bridge Co., Beijing, China). Briefly, tissue slides were dewaxed and endogenous peroxidase was blocked by 3% hydrogen peroxide. After blocking with normal goat serum, primary rabbit anti-human polyclonal antibodies against IL-17 (IL-17A, 1:200 dilution, Santa Cruz, CA, USA) and p-Stat3 (1:400 dilution, Santa Cruz, CA, USA) were applied onto the tissue slides following the procedures as described previously [18].

**Fig 1:**
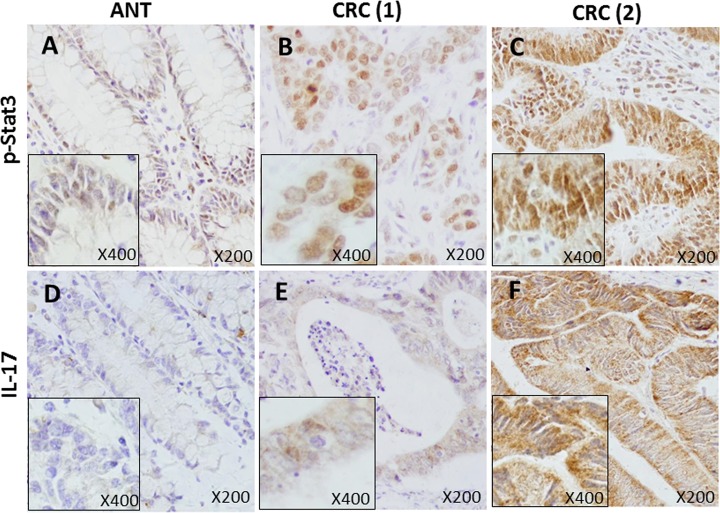
CRC tissues show constitutively over-phosphorylated Stat3 (p-Stat3) and overexpressed IL-17 than adjacent normal tissues (ANT) using IHC method with specific antibodies. Six representative IHC staining panels are shown. Antibody-staining against p-Stat3 is mainly localized in the nucleus (A, B and C, positive staining of yellow-brown), while antibody-staining against IL-17 is primarily in the cytoplasm (D, E and F, positive staining of yellow-brown). A and B came from paired tissues of the same patient but C was from an unrelated tissue. D and E came from paired tissues of the same patient but F was from an unrelated tissue. It can be seen that p-Stat3 is strongly stained in CRC (2) (C, scored as 3+) as compared with another CRC (1) (B, scored as 2+) or ANT tissue (A, scored as negative or 0). Similarly for IL-17, CRC (2) tissue shows strong staining (F, scored as 3+) compared with another CRC (1) (E, scored as 1+) but ANT shows little or no staining (D, scored as negative or 0). Microscopic magnification is X200 with inserts of X400. ANT = adjacent normal tissues and CRC = colorectal cancer, respectively.

Positive IHC stains were defined as yellow-brown color according to the manufacturer’s demonstrative slides (Fig. 1). IHC staining slides were read on an Olympus optical microscope over yellow-brown color stains for 12 consecutive fields and scored according to two variable factors: (1) counting the number of positively stained cells (0 = <5%; 1 = 6%-25%; 2 = 26%-50%; 3 = 51%-75%; and 4 = 76%-100%); and (2) scoring the intensity of the staining (0 = absent; 1 = weak; 2 = moderate; 3 = strong). The final score was the product of (1) multiplying (2) for individual slides (Table 1). This scoring system was similar in principle to a published literature [19]. As tabulated in Table 1, we expressed final staining scores in the following ways for simplicity: negative staining as 0 for (-); incremental positive staining as 1+ for (+); 2+ for (++); and 3+ for (+++), respectively.

**Table 1:** Scoring criteria of Immunohistochemistry (IHC) assay with specific antibodies used in this study.

Staining positive cells		Staining intensity		Final score product	
Percent (%)	Score 1	Intensity	Score 2	Score 1 x Score 2	Score 3
≤5%	0	absent	0	0–1	0 (-)
6%-25%	1	weak	1	2–4	1+ (+)
26%-50%	2	moderate	2	5–8	2+ (++)
51%-75%	3	strong	3	9–12	3+ (+++)
76%-100%	4				

Note: Scoring results are based on screening 12 consecutive microscopic fields. Percent positive cells (score 1) multiply staining intensity (score 2) equals to final product score (score 3). Either 0 or (-) depicts negative staining. For example, an individual slide had <5% of staining cells (= 0) with a staining intensity of 1 (= weak) which would generate a final product score of 0 X 1 = 0; another slide had 80% of staining cells (= 4) with a staining intensity of 3 (= strong) which would give a final product score of 4 X 3 = 12 (3+).

### Statistical Analyses

Statistical analyses were performed using the SPSS statistical software package (version 17.0). χ^2^ test and t-test were adopted for variance analysis and correlation was analyzed using Spearman rank correlation method. Differences with *P*<0.05 were considered statistically significant.

## Results

### HR-HPV Infection Is a Common Event in Chinese CRC Patients

Throughout the entire DNA extraction and HPV testing, we noticed two batches of DNA extracts among which the positive control did not show positivity for HPV and these two batches of 40 tissues were then re-extracted for DNA and re-tested for HPV until the positive HPV control was positive for HPV test. However, we did not observe the negative HPV control turned to be positive through DNA extraction and HPV testing, suggesting an appropriate management of cross-contamination during HPV testing. HPV infection was detected in 46/95 of CRC cases (48.4%, 95% CI 38%-58%) which was much higher than ANT (15/50 or 30.0%, 95% CI 17%-42%) and colorectal adenomas (CRA, 14/50 or 28.0%, 95% CI 16%-40%), respectively (Table 2). Among 46 HR-HPV positive CRC cases, 45 (97.8%) were infected with HPV16 while only one case was infected with HR-HPV subtype, HPV53.

**Table 2:** Colorectal cancer (CRC) tissues have a higher rate of high-risk HPV infection, than both adjacent normal tissues (ANT) and colorectal adenoma tissues (CRA).

Tissue	Total	Presence of HPV16/53 Infection		χ^2^	*P* value
Origins	n	Pos+ n (%, 95% CI)	Neg- n (%, 95% CI)		
CRC	95	46 (48.4, 38–58)	49 (51.6, 42–62)		
ANT	50	15 (30.0, 17–42)	35 (70.0, 83–57)	4.6	0.033
CRA	50	14 (28.0, 16–40)	36 (72.0, 60–84)	5.6	0.018

Note: All HPV-positive tissues/cases were infected by HR-HPV subtypes. Among 46 HPV-positive CRC cases, 45 were HPV16-positive (45/46, 97.8%) and 1 was HPV53-positive (1/46, 2.2%). Furthermore, all 15 HPV-positive ANT tissues (15/15, 100%) and all 14 CRA tissues (14/14, 100%) were infected with HPV16. No other HPV types were detected in all of the above tissues. HPV infections of CRC cases were compared with those of ANT and CRA, respectively. Pos+ = positive; Neg- = negative; CI = confidence interval. CRC, colorectal cancer; ANT, adjacent normal tissues; CRA, colorectal adenoma that included 3 histologic subtypes: tubular adenoma, villous-tubular adenoma, and villous adenoma, among which no differences in HPV infection rate were found (χ^2^ = 1.2, *P* = 0.543).

Furthermore, analyzing the 50 CRC tissues with paired ANT tissues (Table 2) showed that the frequency of HPV infection was also higher in CRC tissues (54%, 27/50) than that in ANT tissues (30%, 15/50, χ^2^ = 5.9, *P* = 0.015). Among these 50 paired cases, 6 cases were HPV16-positive in both CRC and ANT tissues (6 cases, CRC-positive/ANT-positive); 14 cases were HPV-negative in both CRC and ANT tissues (14 cases, CRC-negative/ANT-negative); 21 cases were HPV-positive in CRC but HPV-negative in ANT tissues (21 cases, CRC-positive/ANT-negative, 20 of HPV16-positive and 1 of HPV53-positive); and the remaining 9 cases were HPV16-positive in ANT tissues but HPV16–negative in CRC tissues (9 cases, ANT-positive/CRC-negative). In terms of HPV infection, no correlation was found between the 50 CRC tissues and their paired ANT tissues (r = -0.18, *P* = 0.201). The above observations indicated a fact that HPV infection was a common event in Chinese CRC patients (48.4%), and CRC tissues had a higher frequency of HPV infection than both their paired ANT tissues (χ^2^ = 5.9, *P* = 0.015) as well as unpaired ANT tissues (χ^2^ = 4.6, *P* = 0.033, Table 2). The rate of HPV infection, however, had no difference between 50 ANT tissues and 50 CRA tissues (χ^2^ = 0.05, *P* = 0.826, Table 2).

Two additional observations were interesting: (1) no low-risk HPV subtypes were detected in all of our tissues tested; (2) among all tissues originated from ANT and CRA, we only detected a single HR-HPV16 subtype (Table 2). In addition, all HPV-infected tissues were positive for single HPV subtypes and no tissues were found positive for double or multiple infections of HPV subtypes. Among 50 cases of colorectal adenomas (CRA), no differences in the frequencies of HPV16 infection were noticed among 3 CRA subtypes of tubular, villous-tubular, and villous adenomas (χ^2^ = 1.2, *P* = 0.543, Table 2).

### HPV Infection Correlates with Late Clinical Stages of CRC

To understand whether HPV infection would affect patients’ clinicopathological characteristics, we investigated possible impacts of HPV infection on a number of clinicopathological characteristics including age, gender, tumor location, histological grading, lymphatic metastasis, clinical staging [20] among others. The analyses turned out to show that HPV infection correlated with late or poorer clinical staging of III+IV (Table 3). Furthermore, more detailed analyses of HPV infection against clinical stages in the order of I+II vs III or IV or III+IV showed incremental differences as defined by *P* values (I+II vs III, *P* = 0.058; I+II vs IV, *P* = 0.034; I+II vs III+IV, *P* = 0.028, respectively). Because clinical stages III+IV were defined as having positive metastasis either to lymph node or to other distant organs, the above correlations suggested a possible role of HPV infection in the progression and metastasis of CRC. Among the 50 paired ANT tissues, we failed to observe any correlations between HPV infection and clinicopathological characteristics as shown in Table 3 including cell differentiation, metastasis, clinical staging among others analyzed (data not shown).

**Table 3:** HPV Infection Only Correlates with Late Clinical Stages among Many Clinicopathological Characteristics of CRC.

Characteristics	Total	HPV infection		χ^2^	*P* value
	n	Pos+ n (%)	Neg- n (%)		
Location					
Colon	43	20 (46.5)	23 (53.5)	0.1	0.735
Rectum	52	26 (50.0)	26 (50.0)		
Age					
<60	29	15 (51.7)	14 (48.3)	0.2	0.669
≥60	66	31 (47.0)	35 (53.0)		
Gender					
Men	52	26 (50.0)	26 (50.0)	0.1	0.735
Women	43	20 (46.5)	23 (53.5)		
Differentiation					
Moderately	79	37 (46.8)	42 (53.2)	0.5	0.492
Poorly	16	9 (56.2)	7 (43.8)		
Lymphatic metastasis	
Positive		43	25 (58.1)	18 (41.9)	3.0	0.085
Negative	52	21 (40.4)	31 (59.6)			
Clinical stage					
I+II	86	38 (44.2)	48 (55.8)	4.9	**0.028**
III+IV	9	8 (88.9)	1 (11.1)		
General form					
Ulcerative	75	35 (46.7)	40 (53.3)	1.6	0.665
Protrude	16	8 (50.0)	8 (50.0)		
Infiltrative	3	2 (66.7)	1 (33.3)		
Colloid	1	1 (100)	0 (0)		
Total cases	95	46 (48.4)	49 (51.6)		

Note: CRC, colorectal cancer. Positive cases were compared with negatives, respectively, using chi-square method.

### Constitutively Overactive p-Stat3 and Overexpression of IL-17 Cytokine in CRC

As shown in Fig. 1, p-Stat3 antibody was little stained in ANT tissue (A, scored as 0), however, strongly stained in CRC tissue (C, scored as 3+). Similarly, IL-17 antibody had no staining in ANT tissue (D, scored as 0) but showed strong staining in CRC tissue (F, scored as 3+). Overactive p-Stat3 and overexpressed IL-17 in CRC were apparent when compared with ANT in the whole cohort. As tabulated in Table 4, we compared three categories of positive staining combinations (1+, 2+/3+, and 1+/2+/3+, respectively) with negative staining (0) for p-Stat3 and IL-17, respectively. In general, significantly more CRC tissues showed active p-Stat3 than ANT tissues (1+/2+/3+ vs 0: 95.8% vs 76.7%, *P*<0.001). The differences were particularly obvious for strong active p-Stat3 (2+/3+ vs 0: 50.5% vs 18.9%, *P*<0.001). For IL-17, on the other hand, this phenomenon (see Table 4) was strikingly similar to p-Stat3 in that many more CRC tissues showed strong expression of IL-17 than ANT tissues (2+/3+ vs 0: 45.3% vs 22.2%, *P*<0.001).

**Table 4:** More CRC Tissues Show Strong Positive Stainings (2+/3+) of Constitutive p-Stat3 and Expressed IL-17 than ANT Tissues.

Characteristics	n	Negative	Positive Combinations								
		0 (%)	1+ (%)	χ^2^	*P*	2+/3+ (%)	χ^2^	*P*	1+/2+/3+ (%)	χ^2^	*P*
p-Stat3											
CRC	95	4 (4.2)	43 (45.3)	7.1	0.008	48 (50.5)	24.8	<0.001	91 (95.8)	14.5	<0.001
ANT	90	21 (23.3)	52 (57.8)			17 (18.9)			69 (76.7)		
IL-17											
CRC	95	2 (2.1)	50 (52.6)	7.7	0.006	43 (45.3)	17.4	<0.001	93 (97.9)	11.7	<0.001
ANT	90	15 (16.7)	55 (61.1)			20 (22.2)			75 (83.3)		

Note: Positive combinations are compared with the negative or 0, respectively, using χ^2^ method. CRC, colorectal cancer; ANT, adjacent normal tissues.

**Fig 2:**
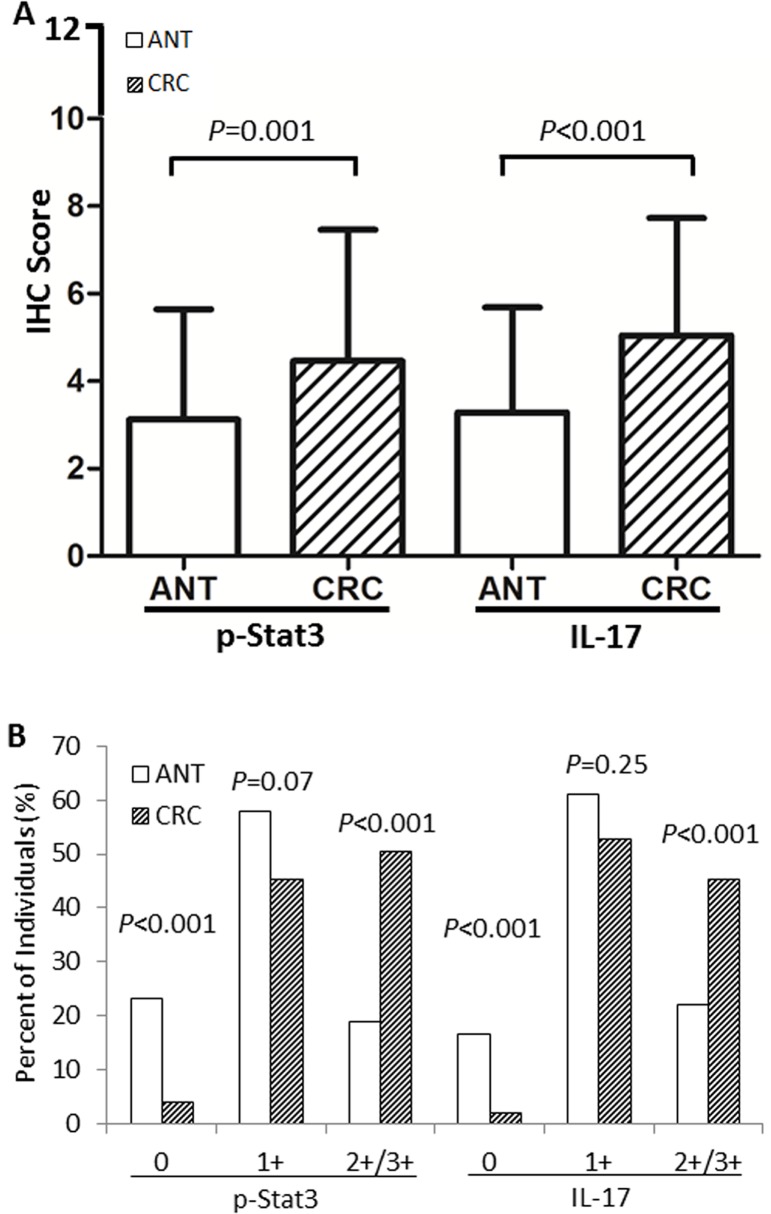
CRC tissues show over-phosphorylation of Stat3 (p-Stat3) and overexpression of IL-17 when compared with ANT. (A) Based on the scoring criteria (Table 1), pooled scores for p-Stat3 or IL-17 are compared between CRC and ANT individuals using t-test and the data are expressed as mean ± standard deviation (M±SD). As seen, constitutive p-Stat3 is higher in CRC than in ANT (M±SD, 4.5±3.0 vs 3.1±2.5, *P* = 0.001). Similarly, expressed IL-17 is also higher in CRC than in ANT (M±SD, 5.0±2.7 vs 3.3±2.4, *P*<0.001). (B) Comparison between CRC and ANT for the number of individuals (%) with varying staining intensities using χ^2^ test. As shown for p-Stat3, CRC category has less individuals scored as 0 than ANT category (4.2% vs 23.3%, *P*<0.001). To the contrary, CRC has many more individuals scored as strong positives (2+/3+) than ANT (50.5% vs 18.9%, *P*<0.001). A striking similarity is true for IL-17, of which CRC category has less individuals scored as 0 than ANT category (2.1% vs 16.7%, *P*<0.001) but again, CRC has many more individuals scored as stronger positives 2+/3+ than ANT (45.3% vs 22.2%, *P*<0.001). For both p-Stat3 and IL-17, staining intensity of weak positivity (1+) is not significantly different between CRC and ANT categories (*P* = 0.07 and *P* = 0.25, respectively). ANT indicates adjacent normal tissues and CRC depicts colorectal cancer, respectively.

To investigate general differences between CRC and ANT for their Stat3 activities and IL-17 levels, we compared pooled IHC scores from all individuals tested for p-Stat3 and IL-17 using t-test. As shown in Fig. 2, panel A, constitutive p-Stat3 was significant higher in CRC tissues than in ANT tissues (M±SD, 4.5±3.0 vs 3.1±2.5, *P* = 0.001). Very similarly, IL-17 levels were also higher in CRC tissues than in ANT tissues (M±SD, 5.0±2.7 vs 3.3±2.4, *P*<0.001). These observations, from a different angle, were supporting the observations as presented in Table 4, indicating an inflamed microenvironment in tumor tissues. On the other hand, panel B in Fig. 2 revealed a phenomenon that CRC in fact had more individual tissues with strong staining (2+/3+ positives) for both p-Stat3 (*P*<0.001) and IL-17 (*P*<0.001), again, in keeping with the observations as shown in Table 4 (2+/3+ positives). The percent of individuals with weak staining (1+ positives), however, was not different between CRC and ANT tissues for either p-Stat3 (*P* = 0.07) or IL-17 (*P* = 0.25).

It was a puzzling phenomenon that we failed to observe any correlations of either p-Stat3 activities or IL-17 levels with clinicopathological characteristics among these CRC patients including cell differentiation, metastasis, clinical staging among others analyzed (Table 5). Furthermore, we also failed to observe any correlations of p-Stat3 activities or IL-17 levels with clinicopathological characteristics among the 50 paired ANT tissues, respectively (data not shown). These results may possibly suggest an early involvement of Stat3 signaling pathway in the pathogenesis of CRC.

**Table 5:** p-Stat3 and IL-17 Expression Are Not Correlated with CRC Patients’ Clinicopathological Characteristics.

Characteristics	n	p-Stat3		χ^2^	*P* value	IL-17		χ^2^	*P* value
		0/1+ (%)	2+/3+ (%)			0/1+ (%)	2+/3+ (%)		
Location									
Colon	43	24 (55.8)	19 (44.2)	1.3	0.261	28 (65.1)	15 (34.9)	3.4	0.065
Rectum	52	23 (44.2)	29 (55.8)			24 (46.2)	28 (53.8)		
Age									
<60	29	15 (51.7)	14 (48.3)	<0.1	0.985	14 (48.3)	15 (51.7)	0.7	0.402
≥60	66	32 (48.5)	34 (51.5)			38 (57.6)	28 (42.4)		
Gender									
Men	52	27 (51.9)	25 (48.1)	0.3	0.600	27 (51.9)	25 (48.1)	0.4	0.545
Women	43	20 (46.5)	23 (53.5)			25 (58.1)	18 (41.9)		
Differentiation									
Moderately	79	38 (48.1)	41 (51.9)	0.4	0.552	43 (54.4)	36 (45.6)	<0.1	0.894
poorly	16	9 (56.2)	7 (43.8)			9 (56.2)	7 (43.8)		
Lymphatic metastasis									
Positive	43	24 (55.8)	19 (44.2)	1.8	0.183	26 (60.5)	17 (39.5)	1.0	0.308
Negative	52	23 (44.2)	29 (55.8)			26 (50.0)	26 (50.0)		
Clinical stage									
I+II	86	41 (47.7)	45 (52.3)	1.8	0.371	45 (52.3)	41 (47.7)	2.1	0.177
III+IV	9	6 (66.7)	3 (33.3)			7 (77.8)	2 (22.2)		
General form									
Ulcerative	75	38 (50.7)	37 (49.3)	1.6	0.837	42 (56.0)	33 (44.0)	1.9	0.647
Protrude	16	7 (43.8)	9 (56.2)			7 (43.8)	9 (56.2)		
Infiltrative	3	1 (33.3)	2 (66.7)			2 (66.7)	1 (33.3)		
Colloid	1	1 (100)	0 (0)			1 (100)	0 (0)		
Total cases	95	47 (49.5)	48 (50.5)			52 (54.7)	43 (45.3)		

Note: Strong positive cases (2+/3+) are compared with weak positive and negative cases (0/1+), respectively, using chi-square method.

### HPV Infection Correlates with p-Stat3 Activities and IL-17 Levels

To investigate whether HPV infection would play a role in orchestrating a tumor environment with heightened active Stat3 and IL-17 levels, we conducted bivariate correlational analyses. In CRC, as shown in Table 6, strong positive correlations were revealed between HPV infection and activated Stat3 levels (r = 0.38, *P*<0.01); between HPV infection and IL-17 levels (r = 0.33, *P*<0.01); and between p-Stat3 and IL-17 levels (r = 0.44, *P*<0.01). However, such correlations among HPV infection, p-Stat3 levels and IL-17 levels were not existent in ANT tissues (r = 0.05, 0.06, and -0.07, respectively, with *P*>0.05).

**Table 6:** Cross correlation analyses reveal strong relationships among HPV infection, activated p-Stat3 and expressed IL-17 in CRC tissues but not in non-cancerous ANT tissues.

Characteristics	HPV	p-Stat3	IL-17
CRC			
HPV	1	0.38**	0.33**
p-Stat3	0.38**	1	0.44**
IL-17	0.33**	0.44**	1
ANT			
HPV	1	0.06	0.05
p-Stat3	0.06	1	- 0.07
IL-17	0.05	- 0.07	1

Note: The numbers shown in the table are correlation coefficient r values. Spearman rank correlation analysis was used.

** indicates *P*<0.01 or otherwise *P*>0.05 for the rest comparisons.

CRC, colorectal cancer; ANT, adjacent normal tissues.

## Discussion and Conclusions

The current paradigm is that the immune system and the microbiota mutually interact to maintain homeostasis in the human intestine. However, components of the microbiota may alter this balance and promote chronic inflammation, further fueling tumor development [4]. Among them, mostly reported are John Cunningham virus (JC virus), BK virus, human cytomegalovirus (CMV), human papillomaviruses (HPV, particularly type 16 and 18) [21].

High-risk HPV (HR-HPV) infection is the major cause for cervical cancer and HPV vaccination is effective in preventing cervical cancer [22]. Interestingly, HPV infection has also been found in many non-cervical cancers. For example, several meta-analyses in colorectal adenomas and adenocarcinomas have reported that, among 2,630 adenocarcinomas stratified by geographical regions, HPV infections are found to be the highest in patients from South America (45.1%), Asia (39.2%), and the Middle East (32.2%) [23,24]. Other meta-analyses have also reported HPV infections in cancers of the ovary [25], bladder [26], lung [27] and breast [28]. These findings indicate a fact that HPV infection is a common event in many types of cancers, suggesting the involvement of HPV in the pathogenesis of non-cervical cancers including colorectal cancer. In our patient population, we have observed a high prevalence of HR-HPV infections in CRC patients (46/95 or 48.4%), among which 45 CRC cases are positive for HPV16 (45/46 or 97.8%) and only one case is infected with HR-HPV53.

In addition, we have also tested HPV infections in other cancers and found quite different frequencies of HPV infection. For example, 10.5% of HPV infection were in breast cancer patients (manuscript submitted); 48.4% in CRC patients (this study); and 90.4% in cervical cancer patients (manuscript in preparation). Our observed frequencies of HPV infection in these cancers are generally in agreement with most published reports, suggesting the quality and reliability of the HPV genotyping method used here.

To the best of our knowledge, this is the highest prevalence of HPV infection observed in Chinese CRC, suggestive of potential preventive HPV vaccination [22] among high-risk individuals in Xinjiang Province where these CRC patients were diagnosed. One convenient approach to prove this hypothesis is to follow up those young girls and boys vaccinated by HPV vaccines to see if they have a reduced incidence of CRC in a prospective long-term epidemiological observation.

There are several proposals as to how HPV infects the rectum and colon. Some view as HPV may possibly travel upward from reproductive tract to the rectum and lower half of descending colon [29]. Others, however, have reported no associations of HPV DNA with tumor sites including ascending, transverse, descending, or sigmoid colon, and rectum [7,30]. A majority of reports propose that HPV may travel through blood circulation to infect the colon and rectum [6,31,32,33,34]. In our CRC patients, we have only observed a correlation of HPV infection with late clinical stage CRC (I+II vs III+IV, 44.2% vs 88.8%, *P* = 0.028), but not with any other clinicopathological features including tumor sites (colon vs rectum) (Table 3). Nevertheless, the route of HPV infection in colorectum is unclear, which needs further investigation. On the other hand, our observation on HPV infection suggests a possibility that, during cancer progression, immunologically and metabolically compromised patients with more advanced stage CRC may induce opportunistic viral replication blast in otherwise latent HPV infection, which may lead to increased copy number of HPV being detected in the tumor site. This opportunistic HPV infection may further trigger the activation of Stat3 signaling and the subsequent expression of proinflammatory cytokines such as IL-17 in the tumor microenvironment.

Indeed, constitutively activated Stat3 (p-Stat3) is significantly increased in CRC tissues than in adjacent normal tissues or ANT (Fig. 1), especially the strong positives of p-Stat3 staining (50.5% in CRC vs 18.9% in ANT, Fig. 2 and Table 4). Coincidentally, the expressed IL-17 follows the steps of p-Stat3 to show a much higher level in CRC than that in ANT (45.3% in CRC vs 22.2% in ANT, Fig. 2 and Table 4). The strong p-Stat3 activities and high IL-17 levels in CRC than in ANT are confirmed by two comparison strategies: one is the differences in pooled IHC scores (Fig. 2A), and the other, the differences in percentages of individual tissues (Fig. 2B). In support of our above observations are studies in other cancers such as ovarian, breast, esophageal, gastric cancers and cutaneous T-cell lymphoma [35,36,37,38,39]. One puzzling observation has been that, unlike HPV infection (Table 3), either p-Stat3 activities or IL-17 levels have failed to correlate with any clinicopathological features of CRC (Table 5). There are two possibilities. One is that HPV-mediated Stat3 activation may only be involved in the initial orchestration of intestinal inflammation and carcinogenesis and then passes the relay to other inflammatory signaling pathways such as NF-κB [4] which may be involved in further developments of CRC. The other may be due to our sample size that is not large enough to pick up differences, if any, among those clinical features. Further investigations would be necessary to clarify these uncertainties.

Taken the above together, in a virus-mediated inflammation, Stat3 signaling pathway may also play a crucial and central role as bacteria do in carcinogenesis [4,11]. Furthermore, the downstream proinflammatory cytokine IL-17 of the Stat3 pathway is likely to acts as an accomplice in orchestrating such a carcinogenic inflammation leading to cancers of the intestine. More importantly, IL-17 also serves as a positive feedback agent to Stat3 signaling and therefore, amplifies the inflammatory status that may further facilitate the formation of CRC.

As mentioned previously, microorganisms may manipulate and/or collaborate with cellular signaling pathways to promote inflammatory microenvironment facilitating cancer development of the intestine. Indeed, we have observed, in CRC but not ANT (Table 6), positive correlations of HPV infection with active p-Stat3 (r = 0.38, *P*<0.01) and with IL-17 levels (r = 0.33, *P*<0.01). Furthermore, it is absolutely relevant that a correlation also exists between p-Stat3 and IL-17 again in CRC (r = 0.44, *P*<0.01), not in ANT (Table 6). This tri-lateral relationship may reveal a possible conspiracy among the three agents, i.e., HPV, Stat3, and IL-17 in promoting CRC. We therefore favor the hypothesis that HPV virus may initiate intestinal inflammation through promoting Stat3 activities (and Stat3-related molecules), which is then sustained by involving Stat3’s downstream cytokines including IL-17, by which creates an inflammatory microenvironment facilitating the development of CRC.

The above notion is echoed by animal studies. For example, in HPV16 E7-infected mice, bacteria can induce a higher level of IL-17 expression than those without E7-infection [40]. Similarly, HPV16 E6 can up-regulate the expression of IL-17 in non-small cell lung cancer [41]. In cervical exfoliated cells, the content of IL-17 in HPV positive patients with cervical cancer is higher than that in HPV negative patients [42]. Additional studies indicate that Stat3 activation is increased in cases of HPV16 positive cervical cancer [43,44]. Furthermore, a mouse model has demonstrated that Stat3 phosphorylation (p-Stat3) in CRC epithelial cells is activated by IL-17, which may be responsible for evolving inflammation into tumor [45]. The expression of T-helper IL-17-producing (Th17) cells is associated with a sharp decline in disease-free survival of early-staged (I/II) colorectal cancers [46], in keeping with our observation that HPV infection is associated with late-staged (III/IV) colorectal cancers (Table 3).

On the other hand, evidence also shows that Stat3 and IL-17 may possess anti-tumor function. For example, IL-17 may trigger anti-tumor responses through producing helper and cytotoxic T cells during tumor development [40,47]. Th17 cells display a late plasticity which is necessary for antitumor activity of Th17 cells [48]. Furthermore, in a murine mammary cancer model, doxorubicin efficiently combines with Th1 or Th17 lymphocytes to suppress tumor development and metastatic disease [49]. Therefore, in terms of cancer development, IL-17 may serve as a “double agent” depending upon a number of conditions including, but not limited to, cancer types, cancer models, and types of dominant immune response (Th1 vs Th2), among others.

In summary, we have demonstrated almost a half of CRC patients infected by HR-HPV, of which 97.8% are HPV16 type, the highest reported thus far in Chinese CRC. HPV infection correlates with poorer clinical stages of CRC suggesting a possible involvement of HPV virus in the full cause of CRC from development to late stage of cancer. For the first time we have revealed a tri-lateral relationship among HPV infection, constitutively activated Stat3 and proinflammatory cytokine IL-17 in human CRC tissues, which implies a possible conspiracy act by which the three agents, possibly with yet to be identified others, collaborate to orchestrate a chronic inflammatory microenvironment that facilitates the development and progression of CRC. Based on the high prevalence of HPV infection in CRC patients, we propose a public health application of prophylactic HPV vaccination that may provide potential benefits among young individuals at least in high-risk regions and/or families with prevalent HPV-associated colorectal cancers.

## Supporting Information

S1 Fig GeneChip hybridization detects HPV DNA in colorectal tissues. Numbers shown in dotted-line boxes represent HPV subtypes (e.g., 16 and 18) and there are 23 HPV-specific probes shown. PC is a positive control (filled blue round dot) placed on every chip membrane. (A) Shows a CRC tissue which is positively hybridized for HPV16 subtype (blue round dot in the square 16). (B) Shows a CRC tissue which is negative for all HPV probe hybridizations.
(TIF)
