# Supplementary figures and images for: Human Papillomavirus Infection Correlates with Inflammatory Stat3 Signaling Activity and IL-17 Level in Patients with Colorectal Cancer
Source: PLoS One. 2015 Feb 23;10(2):e0118391. doi: 10.1371/journal.pone.0118391 (PMC4338045; doi:10.1371/journal.pone.0118391)

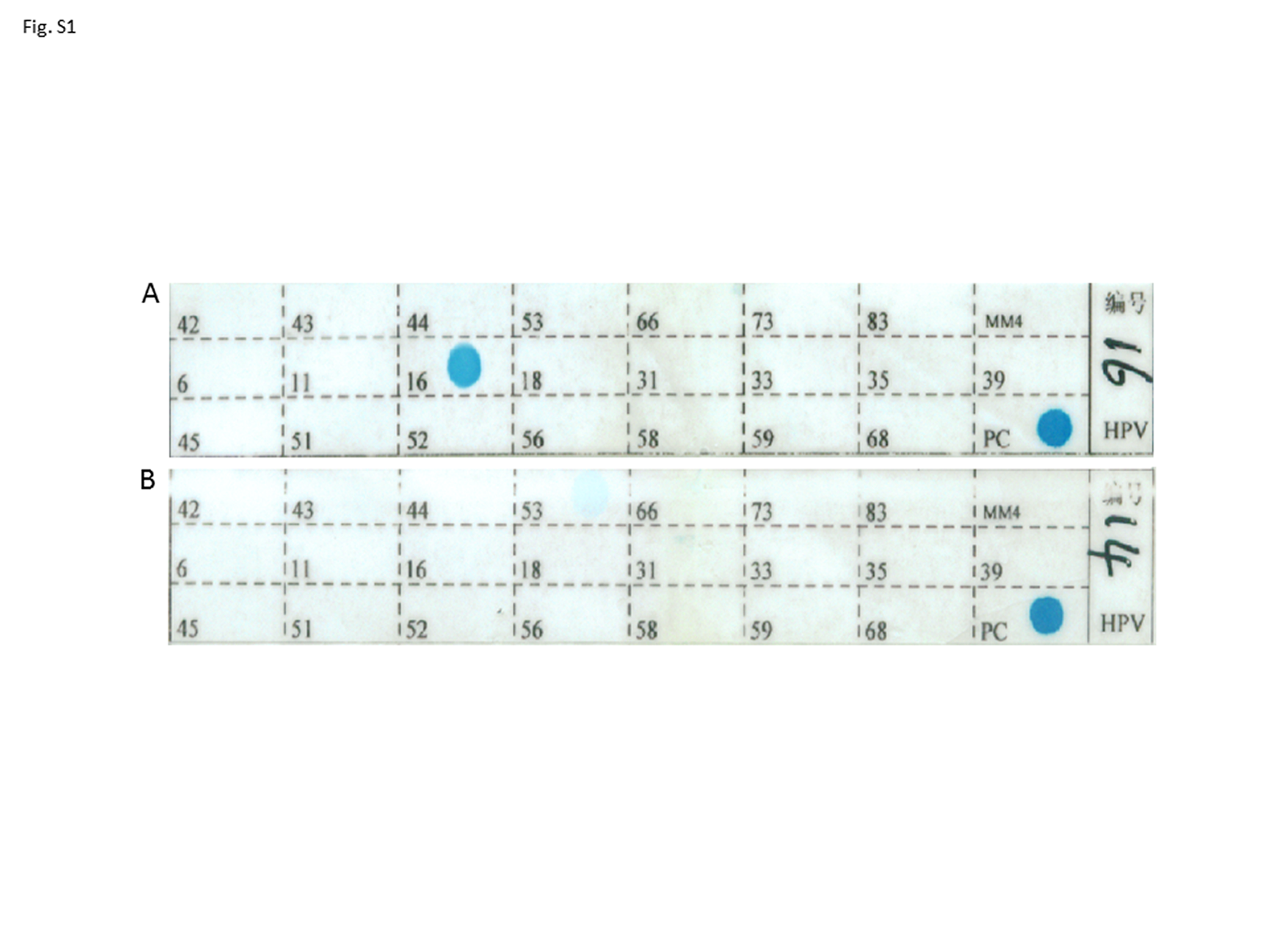

Supplement: S1 Fig — Numbers shown in dotted-line boxes represent HPV subtypes (e.g., 16 and 18) and there are 23 HPV-specific probes shown. PC is a positive control (filled blue round dot) placed on every chip membrane. (A) Shows a CRC tissue which is positively hybridized for HPV16 subtype (blue round dot in the square 16). (B) Shows a CRC tissue which is negative for all HPV probe hybridizations. (TIF) [file pone.0118391.s001.tif]
